# Acute Upper Extremity Arterial Occlusion Diagnosed on POCUS in the Emergency Department 

**DOI:** 10.24908/pocus.v8i1.15902

**Published:** 2023-04-26

**Authors:** Derrick Huang, Jacob Ruzicka, Leoh León, Latha Ganti

**Affiliations:** 1 University of Central Florida College of Medicine/HCA Emergency Medicine Residency Program of Ocala, HCA Florida Ocala Hospital Ocala, FL USA; 2 Emergency Ultrasound Director at Emergency Medicine Residency Program of Ocala, HCA Florida Ocala Hospital Ocala, FL USA; 3 Research Director at Emergency Medicine Residency Program of Ocala, HCA Florida Ocala Hospital Ocala, FL USA

**Keywords:** Point of care ultrasound, Emergency medicine, arterial embolism, upper extremity ischemia, brachial artery occlusion, embolectomy

## Abstract

Upper extremity acute limb ischemia (ALI) is a limb-threatening and potentially lethal pathology that is most commonly caused by vascular embolization. Outcomes of limb ischemia are time-sensitive due to the correlation between a longer time from symptom onset to intervention with a vastly higher risk of amputation. In this report, point of care ultrasound (POCUS) was utilized to rapidly diagnose a patient with a proximal right brachial artery embolic occlusion, prompting expedited surgical consultation and successful embolectomy. POCUS can provide a focused vascular examination of the limbs to expedite diagnosis of time-sensitive ALI and facilitate timely medical intervention and surgical consultation.

## Introduction

Nontraumatic upper extremity acute limb ischemia (ALI) is a limb-threatening and potentially lethal pathology with a rough estimated incidence of about of 1.2 to 3.5 per 100,000 per year, or approximately one-fifth that of acute leg ischemia 1. Upper extremity ALI is most commonly caused by thromboembolism, which is responsible for about 61% of cases, contrasting with lower extremity ALI, which is most commonly caused by thrombosis 2, 3. This phenomenon may be due to the rich network of collateral circulation present in the upper extremities. For example, blood flow can be maintained despite arterial occlusions at the elbow due to collateral vasculature from the radial and ulnar recurrent arteries, deep brachial artery, and dorsal interosseous arteries. Typical causes of thromboembolism in the setting of upper extremity ALI include atrial fibrillation followed by valvular heart disease, left ventricular hypokinesis and aneurysm from coronary heart disease, and thromboembolic sources from proximal arteries 4. In both upper and lower extremities, ALI presents with an onset of symptoms within two weeks and the classic “six Ps” of acute ischemia – pain, pallor, poikilothermia, pulselessness, paresthesia, and paralysis 3.

In the emergency department (ED), assessment of nontraumatic arm pain requires rapid evaluation for causes of ischemia. In addition to embolic and thrombotic causes, these etiologies include post-surgical complications, such as thoracic endovascular aortic repair; post-access complications, such as arterial access for hemodynamic monitoring and arteriovenous access for dialysis; type A aortic dissection; post-neck or upper chest radiation; and various autoimmune diseases 5, 6, 7, 8, 9. Furthermore, non-ischemic etiologies of nontraumatic arm pain, such as rhabdomyolysis, deep vein thrombosis (DVT), and compartment syndrome, can also overlap with ALI. Although point of care ultrasound (POCUS) has been utilized for vascular access and diagnosis of DVTs, this modality has been limited in the evaluation of ALI 10, 11. Here, we present a case of ALI of the upper extremity diagnosed with POCUS requiring emergent vascular intervention. 

## Case

An elderly female with a medical history of atrial fibrillation status post ablation and pacemaker placement (no longer taking anticoagulation due to a history of gastrointestinal bleeding), hypertension, and prior smoking presented to the ED with acute right upper extremity pain and decreased sensation to her right hand with an onset of about 3 hours prior to arrival. The patient reported that the pain involved her upper arm and her hand. She denied similar occurrences in the past. 

**Figure 1:**
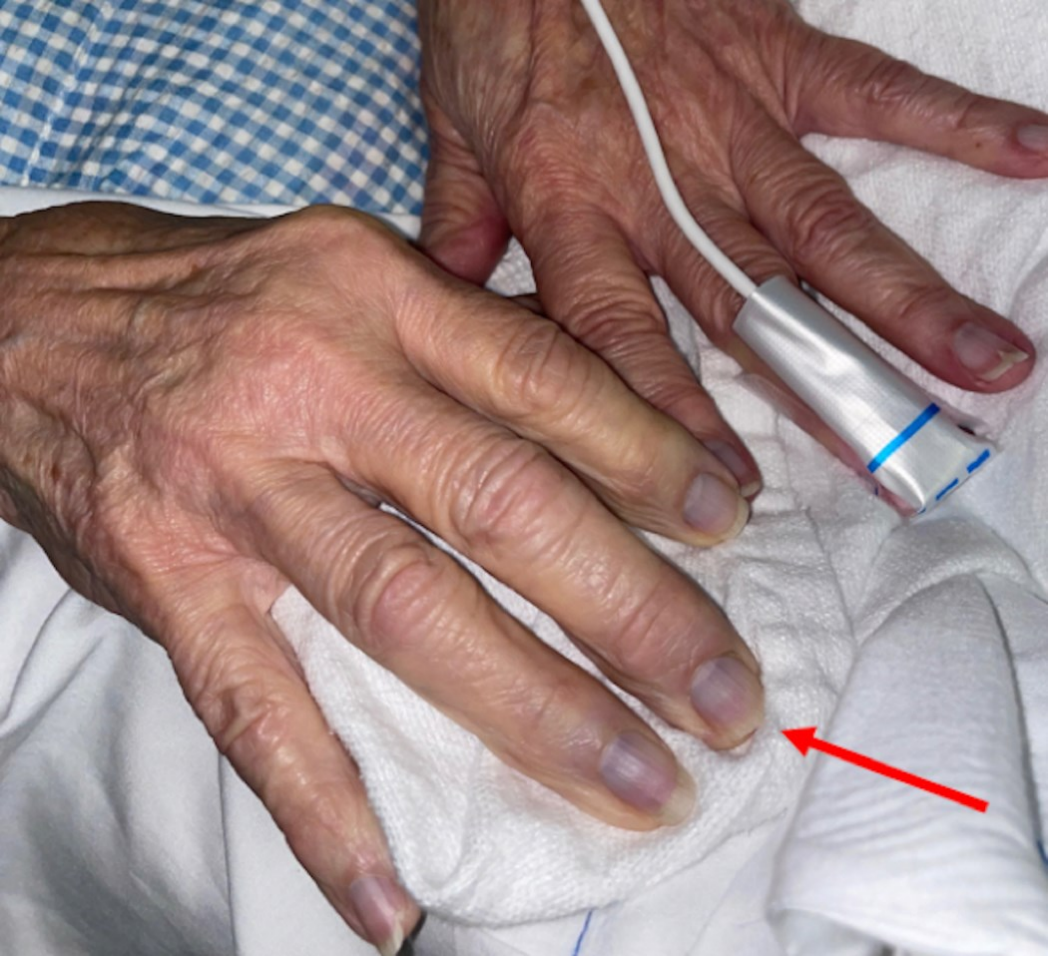
Finger tips of right hand with faint blue discoloration (red arrow).

**Figure 2:**
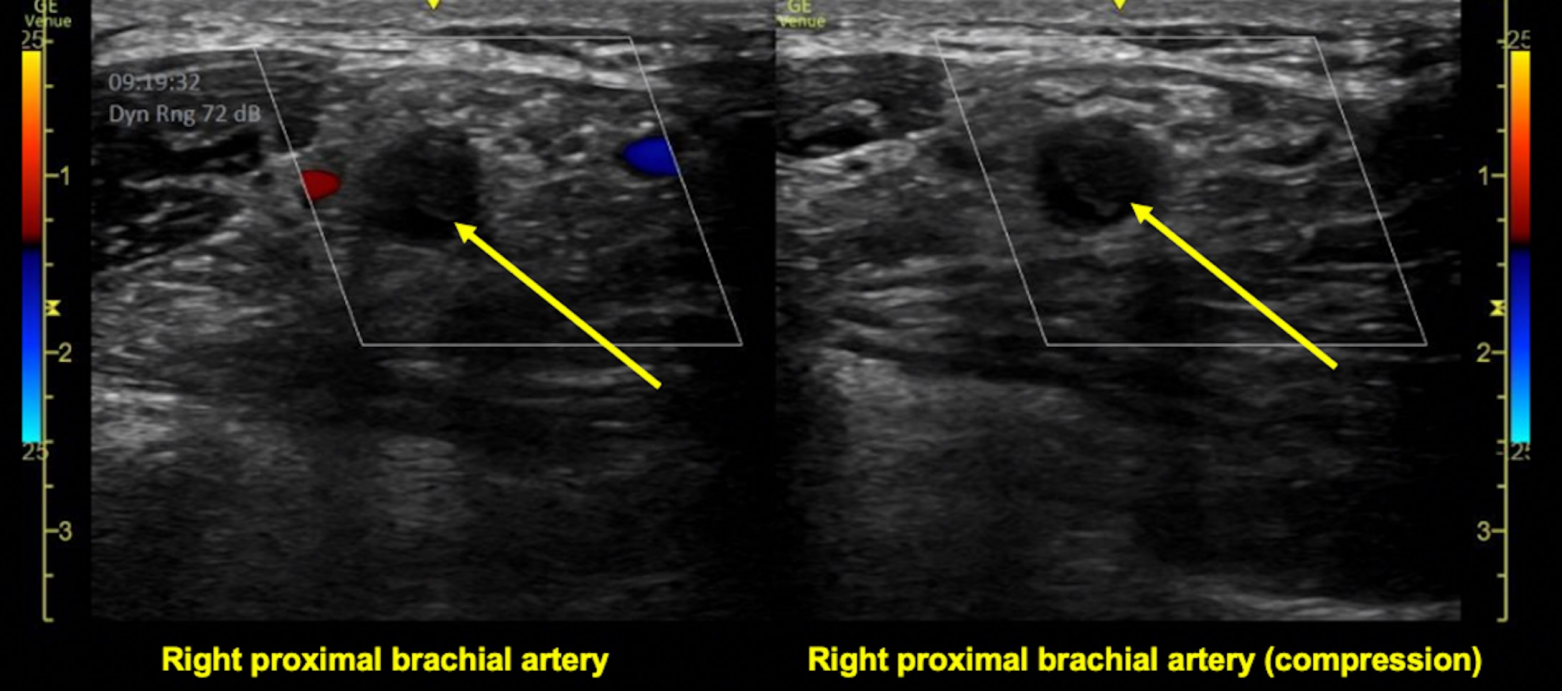
Point of care ultrasound with color Doppler mode in a transverse view utilizing a linear probe to visualize echogenic embolic material (yellow arrow) in the right proximal brachial artery, which was noncompressible and without any Doppler flow signal.

**Figure 3:**
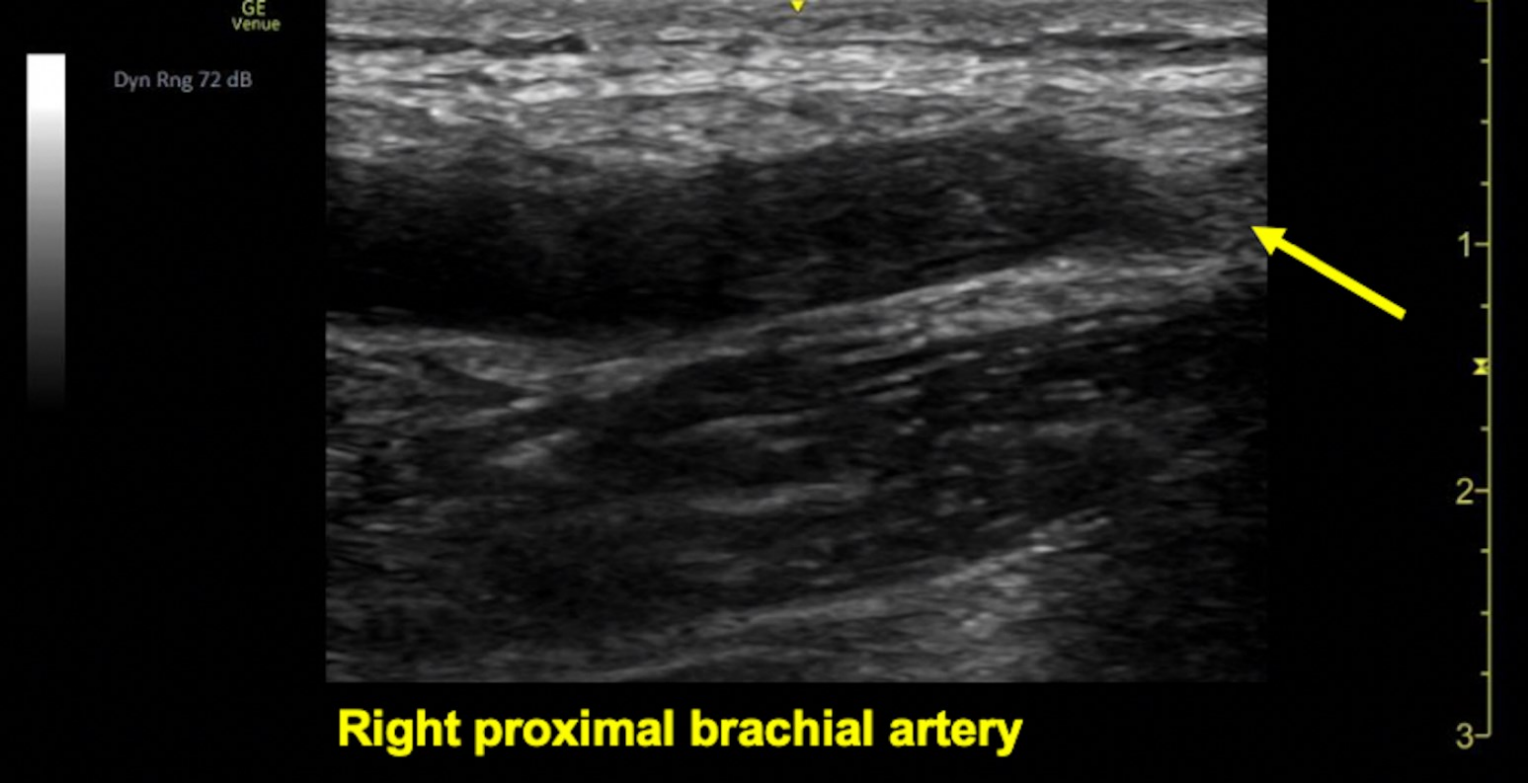
Point of care ultrasound in a longitudinal view utilizing a linear probe to visualize embolic material occluding the lumen of the proximal right brachial artery (yellow arrow).

**Figure 4:**
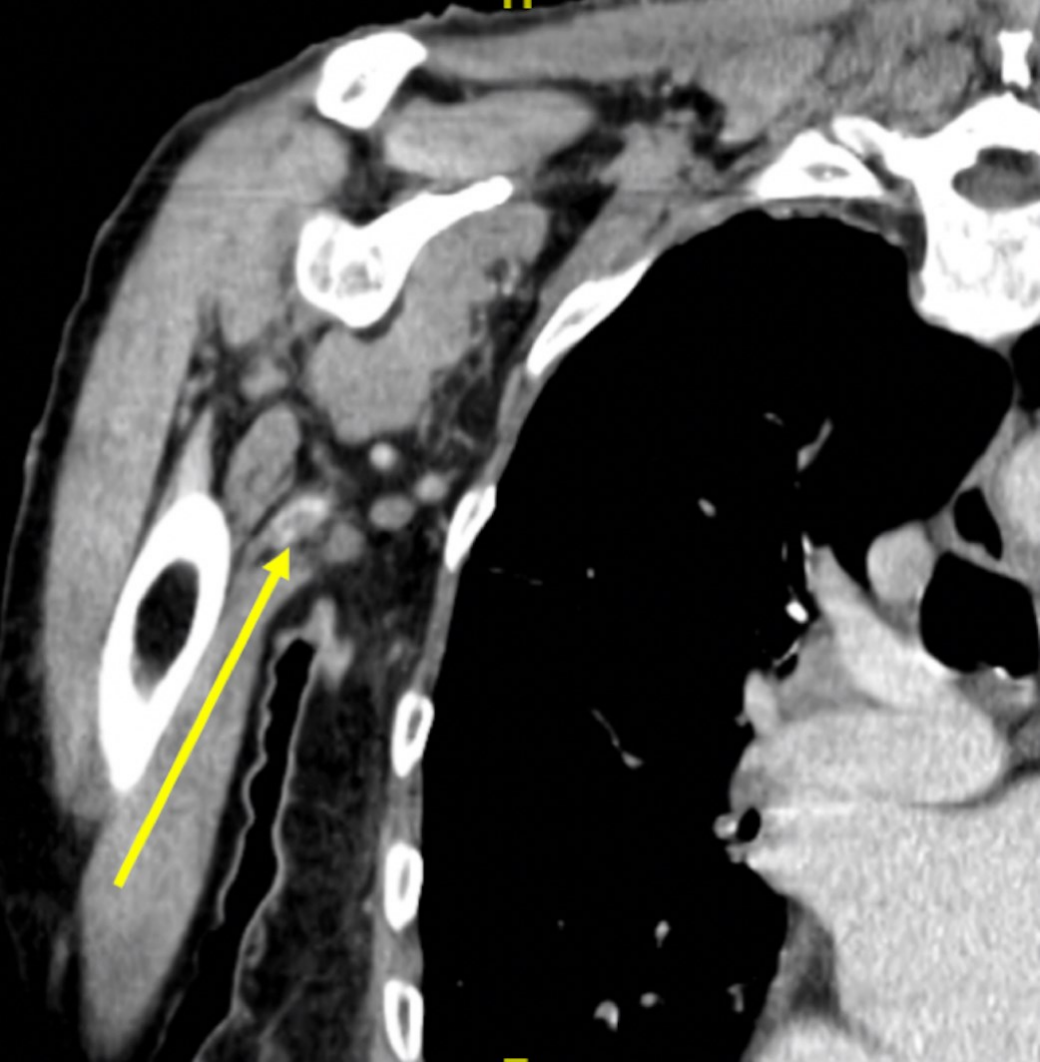
CT angiography of the right upper extremity in the coronal plane. There is a filling defect seen in the proximal right brachial artery at the level of the right humeral neck characteristic of 3 cm embolus (yellow arrow). There is a small amount of flow around this embolus into the mid right brachial artery.

**Figure 5:**
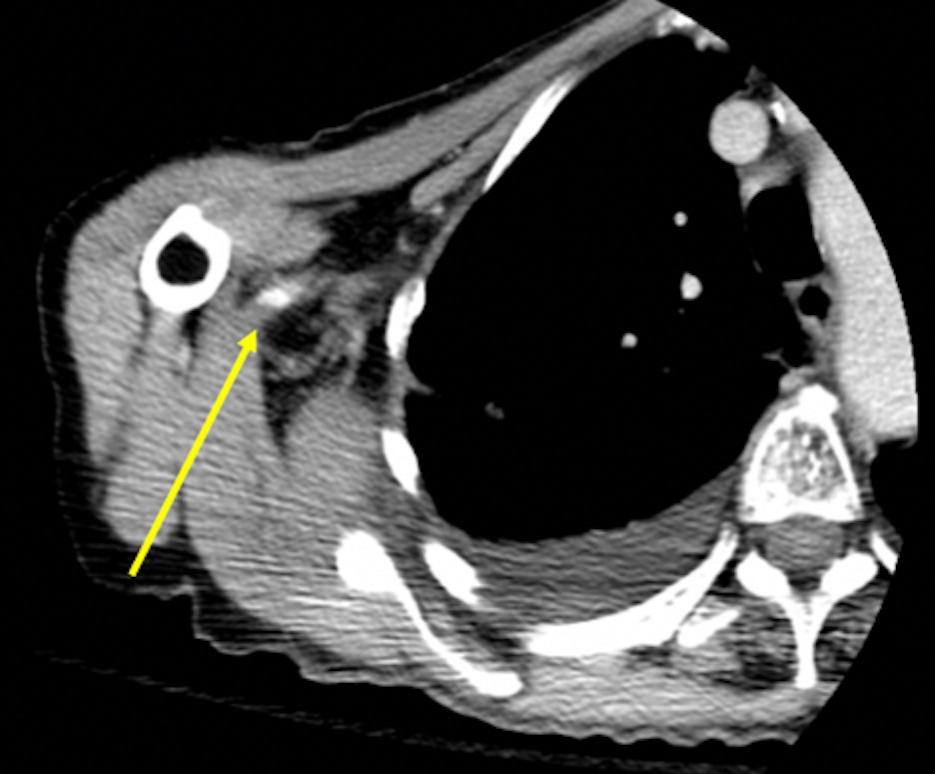
CT angiography of the right upper extremity in the axial plane. There is a filling defect seen in the proximal right brachial artery at the level of the right humeral neck characteristic of embolic debris (yellow arrow).

On arrival, the patient was hemodynamically stable and without distress. She had an irregularly irregular rhythm with a controlled rate. On her right upper extremity exam, the patient had a faint bluish discoloration on her fingers, nonpalpable brachial and radial pulses, 4/5 upper extremity strength, and decreased sensation compared to a normal left upper extremity exam (Figure 1). There was no appreciable edema or erythema. Her laboratory values were unremarkable, including her renal function, coagulation profile, and lactic acid level. POCUS with Doppler was immediately employed and visualized an occlusion at the proximal brachial artery (Figures 2 and 3). Vascular surgery was immediately consulted and a CT angiography (CTA) of the right upper extremity was expedited, showing a 3 cm embolus in the proximal right brachial artery (Figures 4 and 5). Heparin was initiated and the patient was taken to the operating room with vascular surgery for an emergent embolectomy. The patient underwent an uncomplicated embolectomy of her right brachial, radial, and ulnar arteries via arm incision and was ultimately discharged from the hospital. 

## Discussion

We report a case of right upper extremity ALI secondary to an embolism to the proximal right brachial artery that was rapidly diagnosed by POCUS. Diagnosis of ALI requires obtaining a history of symptom progression and recurrence as well as risk factors, which include a history of cardioembolic disease, peripheral arterial disease, diabetes, renal failure, smoking, history of radiation, and prior vascular procedures 3, 5, 6, 7, 8, 12, 13, 14. A vascular exam involving comparison of pulses in all extremities and evaluation for dermatological changes consistent with vascular disease is essential. In a patient with worsening limb ischemia, extremity pain begins distally and progresses proximally, with extremity coolness to touch, pallor, and delayed capillary refill. There may be a “water-hammer” pulse palpated where the embolus occludes the artery 4. Subjective neurological symptoms signal early ischemic nerve damage, whereas pulselessness and motor weakness signal advanced ischemia. Although upper extremity ALI can be a clinical diagnosis, POCUS can assist in evaluation of the wide differential of acute nontraumatic upper extremity pain in equivocal cases. 

Despite the established role of POCUS in assessment of aortic disease and DVT, evaluation of acute ALI with POCUS has been sparsely described in the literature and has yet to be commonly incorporated into clinical guidelines 10, 11, 15. Given that treatment delay of greater than 6 hours from onset to intervention vastly increases the rate of limb amputation, the expediency of POCUS can prove useful in rapid diagnosis 10, 16, 17. In a systematic review and meta-analysis of PAD comparing CTA and duplex ultrasonography in the evaluation of hemodynamically significant stenosis (>50% occlusion), duplex ultrasonography had a lower sensitivity (88% vs 95%) but similar specificity (95% vs 96%) compared to CTA, respectively 18, 19. This underscores the potential and capability of POCUS in evaluating for clinically significant arterial occlusion. Furthermore, POCUS can facilitate diagnosis by identifying small vessel disease or localizing occlusions distal to the wrist by establishing patent proximal arterial vessels. By better localizing the pathology, the emergency provider can focus the differential on autoimmune diseases such as Raynaud's phenomenon, systemic sclerosis, and thromboangiitis obliterans, in addition to thromboembolism and thrombotic etiologies 8, 9, 11, 12. 

Medical treatment of ALI should be initiated promptly after diagnosis and with vascular surgery consultation. Heparin is usually administered to prevent clot propagation, and the patient should be given 325 mg aspirin as well as medication for pain control. Generally, embolic occlusions require an embolectomy, whereas thrombotic occlusions are treated with arterial bypass or endarterectomy; however, the choice of intervention is determined by the vascular team in conjunction with the patient's clinical presentation, comorbidities, and etiology 20. The Rutherford classification for ALI can be utilized in risk stratification 21. Here, ALI is categorized as Viable (class I), Threatened (class II), Marginally threatened (IIa), Immediately threatened (IIb), and Irreversible (class III) based on the vascular exam, Doppler findings, and sensorimotor deficits. The provider should be cognizant that muscle weakness portends severe ischemia and progression from class I/IIA to IIB/III, which may prompt disposition towards a more invasive open operative intervention versus an endovascular intervention 20, 21. For class III, the recommended intervention is primary amputation. In less severe presentations of symptomatic upper extremity occlusion with positive Doppler signals, a wrist-brachial index can be obtained for risk stratification 22. In our case, diagnosis and medical intervention was expedited through use of POCUS and the patient was categorized as Immediately threatened (IIb), ultimately prompting emergent endovascular intervention. 

The requirement of proficiency in Doppler physics in both color and pulsed-wave Doppler parameters can be a barrier to more widespread clinical practice of POCUS in the assessment of ALI. These skills are essential for accurate acquisition and interpretation of sonographic images and range from effective use of color box, gain, and velocity scale to understanding Doppler angle and inversion 23. Fortunately, these skills have significant overlap with commonly employed POCUS techniques in the setting of DVT and echocardiography. For example, bedside echocardiography with Doppler can assess for the presence of tricuspid regurgitation that may correlate with increased right ventricular pressure in the setting of a pulmonary embolism 24. This overlap between sonographic evaluation of different pathologies might facilitate training and further use of POCUS in vascular assessment.

## Conclusion

Nontraumatic upper extremity ALI is a time-sensitive, limb-threatening pathology. Given the expediency, ability to localize occlusions, and assessment for flow with POCUS with Doppler, this bedside imaging modality has a significant role in the evaluation of nontraumatic arm pain and rapid diagnosis of ALI. Use of POCUS can facilitate timely diagnosis and medical intervention as well as surgical consultation for definitive vascular 

## Consent

Written consent was obtained from the patient for publication of this case report including images. 

## Conflicts of Interest

All authors declare that they have no conflicts of interest. No financial or material support was provided to the authors. 
